# Cytotoxic Effects of Nanoliposomal Cisplatin and Diallyl Disulfide on Breast Cancer and Lung Cancer Cell Lines

**DOI:** 10.3390/biomedicines11041021

**Published:** 2023-03-27

**Authors:** Kaavya Gunasekaran, Bala Murali Krishna Vasamsetti, Priyadharshini Thangavelu, Karthi Natesan, Bonaventure Mujyambere, Viswanathan Sundaram, Rama Jayaraj, Yeon-Jun Kim, Suja Samiappan, Jae-Won Choi

**Affiliations:** 1 Department of Biochemistry, Bharathiar University, Coimbatore 641046, India; 2 Department of Biomedical Science, Cheongju University, Cheongju 28160, Republic of Korea; 3 Department of Biochemistry, School of Allied Health Sciences, REVA University, Bengaluru 560064, India; 4 Bharathiar Cancer Theranostics Research Center-RUSA-2.0, Bharathiar University, Coimbatore 641046, India; 5 Northern Territory Health and Support Services, Northern Territory Institute of Research and Training, Darwin, NT 0810, Australia; 6 Jindal Institute of Behavioral Sciences (JIBS), Jindal Global Institution of Eminence Deemed to Be University, Sonipat 131001, India; 7 Director of Clinical Sciences, Northern Territory Institute of Research and Training, Darwin, NT 0909, Australia; 8 Department of Biopharmaceutical Sciences, Cheongju University, Cheongju 28160, Republic of Korea

**Keywords:** nanoliposome, cisplatin, diallyl disulfide, anti-cancer therapy

## Abstract

Dual drug delivery has become the choice of interest nowadays due to its increased therapeutic efficacy in targeting the tumor site precisely. As quoted in recent literature, it has been known to treat several cancers with an acute course of action. Even so, its use is restricted due to the drug’s low pharmacological activity, which leads to poor bioavailability and increases first-pass metabolism. To overcome these issues, a drug delivery system using nanomaterials which would not only encapsulate the drugs of interest but also carry them to the target site of action is needed. Given all these attributes, we have formulated dual drug-loaded nanoliposomes with cisplatin (cis-diamminedichloroplatinum(II) (CDDP)), an effective anti-cancer drug, and diallyl disulfide (DADS), an organosulfur compound derived from garlic. The CDDP and DADS-loaded nanoliposomes (Lipo-CDDP/DADS) exhibited better physical characteristics such as size, zeta potential, polydispersity index, spherical shape, optimal stability, and satisfactory encapsulation percentage. The in vitro anti-cancer activity against MDA-MB-231 and A549 cell lines revealed that Lipo-CDDP/DADS showed significant efficacy against the cancer cell lines, depicted through cell nucleus staining. We conclude that Lipo-CDDP/DADS hold exceptional pharmacological properties with better anti-cancer activity and would serve as a promising formulation to treat various cancers.

## 1. Introduction

Cancer therapies include conventional radiation, chemotherapy, and surgery. Conventional chemotherapy involves the administration of a single drug at different dose levels for a limited period. Even though conventional therapy is said to be effective, it gives a serious implication of drug resistance in due course of time. Co-delivery/combination therapy of anti-cancer drugs has been in use for quite a long time now, as it increases the drug efficacy and bioavailability [1]. Moreover, combination therapy can precisely target multiple pathways rather than a single drug therapy which would target a sole way that might later become impossible due to resistance [2,3].

Cisplatin (cis-diamminedichloroplatinum(II) (CDDP)) is a platinum-based drug that is widely used for treating aggressive cancer types such as ovarian cancer, cervical cancer, breast cancer, head and neck cancer, esophageal cancer, lung cancer, mesothelioma, neuroblastoma, etc. [4]. The CDDP traditionally damages the nuclear DNA by forming DNA adducts, resulting in cell senescence and further cell death. Though CDDP is very potent, its use is often limited due to its notable toxic effects, such as nephrotoxicity, hepatotoxicity, and ototoxicity [5,6]. Diallyl disulfide (DADS) is an organosulfur compound derived from garlic. It is said to possess various potential health benefits such as anti-inflammatory, anti-bacterial, and anti-fungal, and it acts as detoxifying agent [7]. It also acts as an immune booster which actively improves immune health. Recent studies have quoted that garlic derivatives can alleviate toxicities induced by CDDP through various signaling pathways [8].

Nanoliposomal therapy has been used actively for years for the delivery of drugs to the site of the target. It has also improved the stability and enhanced the storage life of the drug it carries. Due to their nanoscale size, they act as a perfect cargo for delivering drugs to a particular target site. Due to their bilayer structure (both hydrophilic core and lipophilic layers), encapsulation of hydrophilic and lipophilic drugs becomes uncomplicated [9,10]. Further, various significant characteristics of nanocarriers lead to modifications such as ligand targeting, receptors targeting for a particular pathway, and pH-sensitive modifications for efficient targeting and active delivery of drugs [11]. In addition to improving effective targeting, these modifications trigger tumor-specific immune cells, improving antitumor immunity [12].

Taking these findings and the property of the drugs into consideration, it is believed that the dual drug-loaded nanoliposomes would act as a good delivery tool to transport the drugs efficiently to the target site and reduce the toxicity exerted by CDDP [13]. The present study was thought up to assess the possible therapeutic action of novel dual drug-loaded nanoliposomes on breast cancer cell line (MDA-MB-231) and lung cancer cell line (A549) in vitro.

## 2. Materials and Methods

### 2.1. Materials

The CDDP, DADS, 1,2-dilauroyl-sn-glycero-3-phosphocholine (DLPC), and cholesterol were obtained from Sigma-Aldrich (St. Louis, MO, USA). In addition, Dulbecco’s modified eagle’s medium (DMEM), fetal bovine serum (FBS), L-glutamine, antibiotics, 3-[4,5-dimethylthiazol-2-yl]-2,5-diphenyltetrazolium bromide (MTT), dialysis bag (MWCO: 12,000–14,000), phosphate-buffered saline (PBS) were procured from HiMedia Laboratories (Kelton, PA, USA). All chemicals and solvents used were of analytical grade.

### 2.2. Cell Culture

The MDA-MB-231, A549, HEK-293, and L929 cell lines were obtained from the National Centre for Cell Science (NCCS), Pune, and cultured in DMEM medium supplemented with 10% FBS (*v*/*v*), 1% antibiotics (penicillin/streptomycin) (*v*/*v*) with 5% CO_2_ at 37 °C in an incubator.

### 2.3. Preparation and Characterization of Nanoliposome Formulation

The preparation of nanoliposome-loaded CDDP and DADS was undertaken by thin film hydration method to achieve the best formulation concerning the size, zeta potential, drug entrapment, and release rate. The protocol was carried out as mentioned by Zhang [14] with slight modifications. The mean diameter of small unilamellar vesicles was determined by the dynamic light scattering (DLS) technique. The samples were also measured three times. The zeta potential of nanoliposomes was measured using a Zetasizer Nano ZS90 from Malvern Panalytical (Malvern, UK).

### 2.4. Encapsulation Efficiency

The encapsulation efficiency of CDDP-loaded nanoliposomes (Lipo-CDDP) and DADS-loaded nanoliposomes (Lipo-DADS) was determined by dissolving Lipo-CDDP and Lipo-DADS (1 mL) in isopropanol and centrifuging at 10,000 rpm for 30 min. The supernatant was filtered and estimated for drug concentration using a microplate reader from Thermo Fisher Scientific (Multiskan Go, Waltham, MA, USA) at 212 nm (CDDP) and 290 nm (DADS), respectively. The percentage encapsulation efficiency was determined by the following Equation (1):

Encapsulation efficiency (%) = [Entrapped drug concentration/Total drug concentration] × 100(1)

### 2.5. Physical Characterization of Liposomes

A transmission electron microscope (TEM) from FEI Company (Tecnai Sprit G2, Hillsboro, OR, USA) was used to determine the shape and structure of the CDDP and DADS-loaded nanoliposomes. To prepare the samples for the analysis of TEM, a certain amount of nanoliposome solution was placed on the carbon-coated copper grid, and the solvent was evaporated. In the next stage, the copper grid was positioned in the specimen holder, and the samples were analyzed.

### 2.6. Drug Release of Liposomal Formulation

To examine the drug release profile of CDDP and DADS from nanoliposomes, 10 µL of free CDDP, free DADS, Lipo-CDDP, Lipo-DADS, and Lipo-CDDP/DADS were mixed with 990 µL of ethanol: water (70:30, *v*/*v*) separately and put into a dialysis bag with 50 mL PBS as sink medium. Subsequently, the buffer solutions around the dialysis bag were collected at different time intervals of 0, 0.5, 2, 4, 6, 8, 10, 12, 16, 18, 24, 28, 32, 36, 40, 44 and 48 h and replaced with the same volume of PBS simultaneously. The collected solutions were analyzed at 212 nm (CDDP) and 290 nm (DADS), respectively, using the Multiskan Go microplate reader, and the percentage of drug release was calculated using the following Equation (2):

Percentage of drug release (%) = [Concentration of drug released/Concentration of total drug] × 100(2)

### 2.7. Colloidal Stability

To assess the stability of nanoliposomes, the formulations were stored at 4 °C for three months. The size, zeta potential, polydispersity index (PDI), and encapsulation efficiency of nanoliposomes were assessed after the end of three months.

### 2.8. Cytotoxicity Assay

MDA-MB-231 and A549 cells were seeded in 96-well plates at a density of 5,000 cells per well and incubated overnight. Free CDDP, free DADS, Lipo-CDDP, Lipo-DADS, and Lipo-CDDP/DADS solution were prepared with DMEM medium without FBS and added to the cells at various concentrations. After cells were incubated for 24 h, the media was discarded, and 20 μL MTT reagent was added in each well and incubated for 5 h. Then 100 μL solubilization solution was added to the medium. The absorbance of the colored solution was quantified by a microplate reader at 570 nm. Under the same experimental conditions, normal HEK-293 and L929 cells were used as controls to compare the cytotoxicity of the free drugs and nanoformulations on non-cancerous cells. Half maximal inhibitory concentration (IC_50_) was derived from GraphPad Prism. Inhibition of cell viability was calculated by Equation (3):

% of Inhibition = [(OD of the individual test group) − (OD of the control group)/OD of control group] × 100(3)

### 2.9. Morphological Analysis of MDA-MB-231 and A549 Cell Lines

Briefly, MDA-MB-231 and A549 cells were cultured on 6-well plates with a density of 1 × 10^5^ cells/well and were incubated according to the IC_50_ range of Lipo-CDDP, Lipo-DADS, and Lipo-CDDP/DADS for 24 h. The morphological changes were evaluated by an inverted microscope from Nikon (ECLIPSE Ti2, Tokyo, Japan).

### 2.10. Cell Nucleus Staining

Cytotoxicity exerted by Lipo-CDDP, Lipo-DADS, and Lipo-CDDP/DADS was concluded with 4′,6-diamidino-2-phenylindole (DAPI) staining. MDA-MB-231 and A549 cells were cultured in 6-well plates for 24 h and treated with IC_50_ value of the drugs, followed by staining with DAPI solution. After staining, the cells were visualized by the inverted fluorescence microscope from Nikon.

### 2.11. Statistical Analysis

All statistical data shown were expressed as mean ± standard deviation, and a *t*-test was performed to identify the significance between formulations. The * *p* ≤ 0.05 and ** *p* ≤ 0.01 were statistically significant.

## 3. Results

### 3.1. Determination of Size, Zeta Potential, and PDI

**Figure 1:**
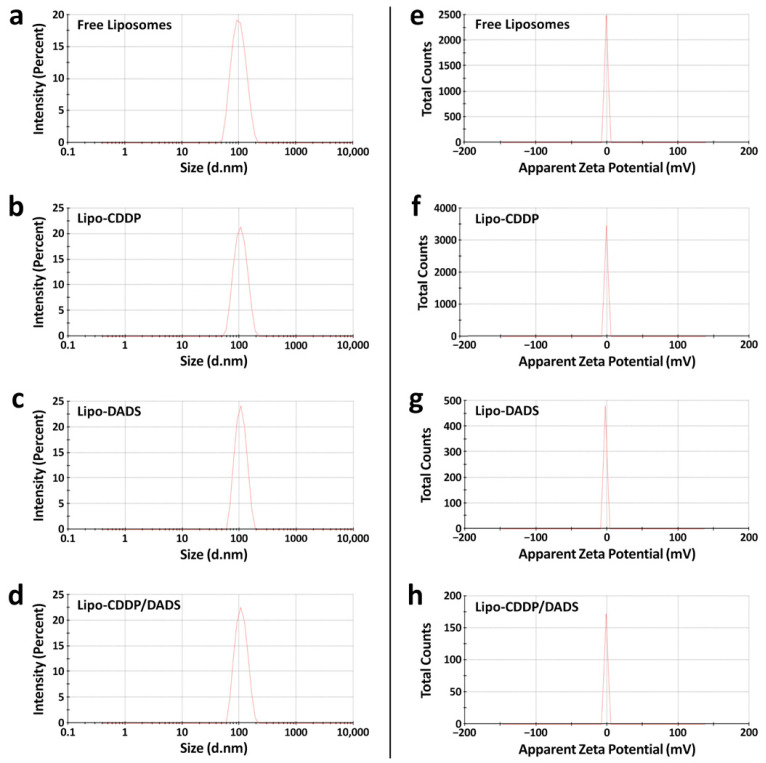
Size profiles of (**a**) free liposomes, (**b**) Lipo-CDDP, (**c**) Lipo-DADS, and (**d**) Lipo-CDDP/DADS and zeta potential profiles of (**e**) free liposomes, (**f**) Lipo-CDDP, (**g**) Lipo-DADS, and (**h**) Lipo-CDDP/DADS. The d.nm in (**a**–**d**) means diameter in nanometers, and the mV in (**e**–**h**) means millivolt.

The liposomal formulations of CDDP and DADS were prepared by the thin film hydration method. The molar ratios of lipid: cholesterol: CDDP: DADS selected for this study was 10:2:1:1. Free liposomes, Lipo-CDDP, and Lipo-DADS showed particle size < 200 nm, i.e., 94.39 nm, 101.60 nm, and 103.40 nm, respectively (Figure 1a–c). Similarly, dual drug-loaded liposomes (Lipo-CDDP/DADS) have shown a particle size of 105.50 nm (Figure 1d and Table 1).

The zeta potential of free liposome, Lipo-CDDP, and Lipo-DADS were −1.01 mV, −1.13 mV, and −2.36 mV, respectively (Figure 1e–g). Similarly, the zeta potential of Lipo-CDDP/DADS was found to be −1.34 mV (Figure 1h). In our study, the PDI values for free liposomes, Lipo-CDDP, Lipo-DADS, and Lipo-CDDP/DADS were found to be 0.088, 0.038, 0.015, and 0.006 (Table 1). This explains the monodispersed characteristics of formulated nanoliposomes throughout the solution.

### 3.2. Encapsulation Efficiency

The encapsulation efficiency of Lipo-CDDP and Lipo-DADS were found to be 90.21 ± 4.21% and 93.14 ± 2.50%. The encapsulation efficiency of Lipo-CDDP/DADS was found to be 80.24 ± 2.32% for CDDP and 89.02 ± 3.50% for DADS, respectively. Being lipophilic, DADS shower higher encapsulation efficiency compared to its hydrophilic (CDDP) counterpart (Table 2).

### 3.3. TEM Analysis

**Figure 2:**
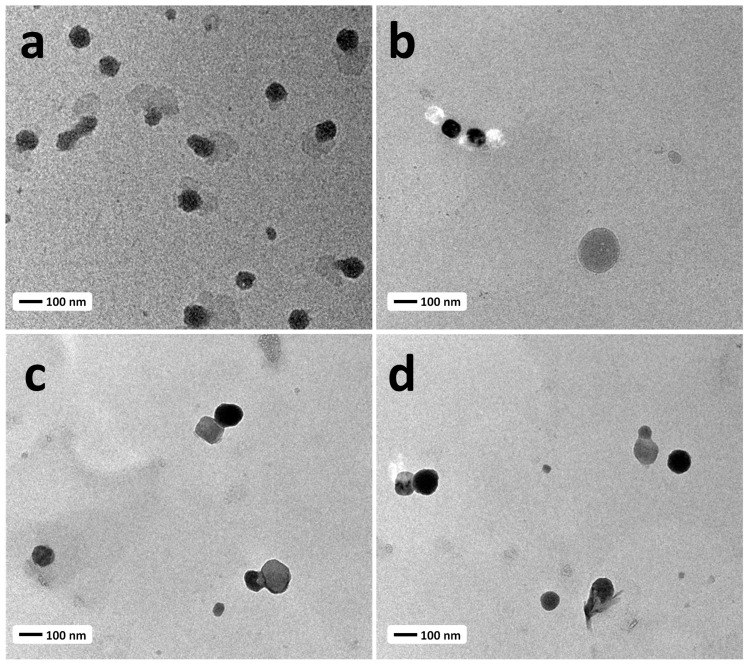
Transmission electron microscopy images of (**a**) Free liposomes, (**b**) Lipo-CDDP, (**c**) Lipo-DADS, and (**d**) Lipo-CDDP/DADS.

TEM images of free liposome, Lipo-CDDP, Lipo-DADS, and Lipo-CDDP/DADS indicate that the liposomes were near spherical with even distribution. There was no significant difference between the free liposomes and Lipo-CDDP/DADS except for a little difference in the size of the formulation (Figure 2a–d). The size of the liposomes was similar to that of the size predicted in the DLS analysis. Further, no significant aggregation of lipids was to be found, which reveals its monodispersity as denoted in PDI.

### 3.4. Drug Release

**Figure 3:**
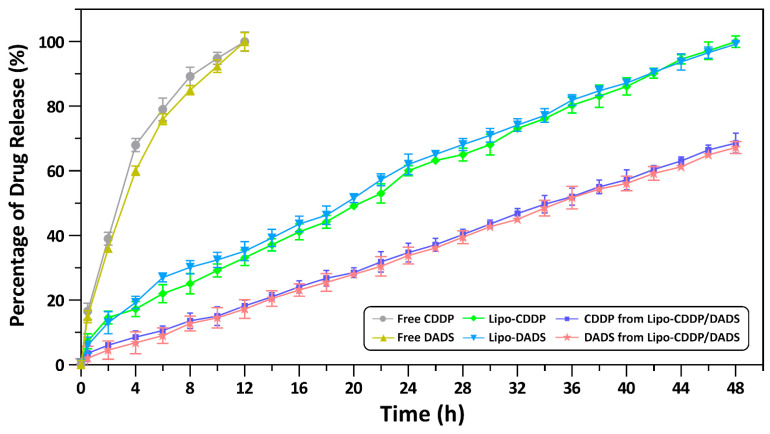
The drug release profile of free drugs and drug(s)-loaded nanoliposomes.

Drug release profiles of free drugs and nanoliposomal formulations were analyzed by the dialysis bag method. The release profile for 48 h of free CDDP, free DADS, Lipo-CDDP, Lipo-DADS, and Lipo-CDDP/DADS showed drastic changes in the release profile. A 100% drug release was observed for free CDDP and free DADS during 12 h. In contrast, Lipo-CDDP and Lipo-DADS showed significant, sustained drug release lasting during 24 h with the drug release rate of 60.42 ± 1.60% and 62.16 ± 3.20%, respectively. Lipo-CDDP/DADS showed a significant and sustained release of 68.59 ± 3.10% for CDDP and 67.23 ± 1.80% for DADS after 48 h (Figure 3). This percentage of release denotes the sustained release of drugs from Lipo-CDDP/DADS, which denotes their stability and efficacy.

### 3.5. Colloidal Stability

**Figure 4:**
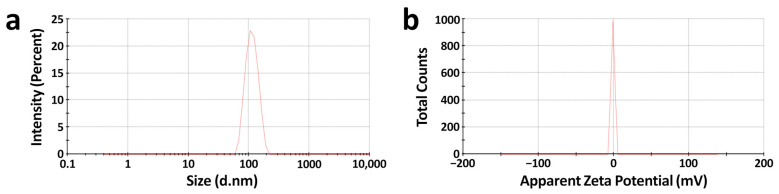
Colloidal stability of Lipo-CDDP/DADS after 90 days. (**a**) Average size and (**b**) zeta potential of Lipo-CDDP/DADS. The d.nm in (**a**) means diameter in nanometers, and the mV in (**b**) means millivolt.

Colloidal stability results revealed that the nanoliposomal formulation remained stable for three months. In addition, no indicative changes in size, zeta potential, PDI, and encapsulation efficiency were observed (Figure 4a,b and Table 3).

### 3.6. Cytotoxicity Analysis

**Figure 5:**
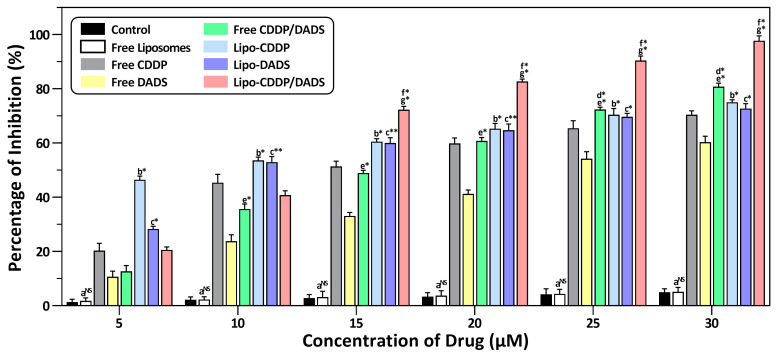
In vitro cytotoxic effect by control, free drugs, and drug(s)-loaded nanoliposomes on MDA-MB-231 cell line. Results are shown as mean ± standard deviation (*n* = 3). Group a, control vs. free liposome; Group b, free CDDP vs. Lipo-CDDP; Group c, free DADS vs. Lipo-DADS; Group d, free CDDP vs. free CDDP/DADS; Group e, free DADS vs. free CDDP/DADS; Group f, Lipo-CDDP vs. Lipo-CDDP/DADS; Group g, Lipo-DADS vs. Lipo-CDDP/DADS. * *p* ≤ 0.05, significance; ** *p* ≤ 0.01, most significance; NS, non-significance.

**Figure 6:**
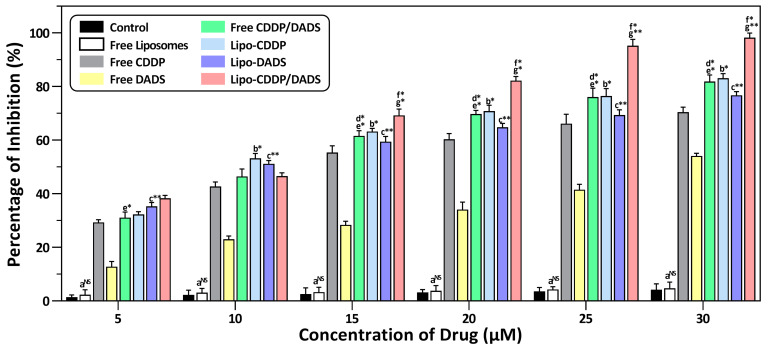
In vitro cytotoxic effect by control, free drugs, and drug(s)-loaded nanoliposomes on A549 cell line. Results are shown as mean ± standard deviation (*n* = 3). Group a, control vs. free liposome; Group b, free CDDP vs. Lipo-CDDP; Group c, free DADS vs. Lipo-DADS; Group d, free CDDP vs. free CDDP/DADS; Group e, free DADS vs. free CDDP/DADS; Group f, Lipo-CDDP vs. Lipo-CDDP/DADS; Group g, Lipo-DADS vs. Lipo-CDDP/DADS. * *p* ≤ 0.05, significance; ** *p* ≤ 0.01, most significance; NS, non-significance.

Cytotoxicity assay was performed to evaluate the effect of all drug formulations on MDA-MB-231 and A549 cell lines, respectively. The IC_50_ of free CDDP and free DADS at 24 h was 11.71 ± 1.50 μM and 24.12 ± 1.20 μM for the MDA-MB-231 cell line, 13.24 ± 1.21 μM and 29.51 ± 0.98 μM for A549 cell line. The IC_50_ for free CDDP/DADS was found to be 15.78 ± 2.02 μM and 11.25 ± 1.85 μM for the MDA-MB-231 cell line and A549 cell line. Similarly, the IC_50_ for Lipo-CDDP and Lipo-DADS were 5.74 ± 0.96 μM and 9.51 ± 1.30 μM for the MDA-MB-231 cell line, and in the A549 cell line, the IC_50_ were 6.25 ± 1.45 μM and 10.21 ± 2.21 μM respectively. Further, the IC_50_ values for Lipo-CDDP/DADS in the MDA-MB-231 cell line were found to be 12.15 ± 0.45 μM, and in A549 the IC_50_ was found to be 14.53 ± 1.67 μM (Figure 5 and Figure 6) as depicted in Table 4. These results show that Lipo-CDDP/DADS showed better cytotoxic activity against both cell lines, even with low drug concentration, than the other formulations. In addition, cytotoxicity tests using normal (non-cancerous) cells, such as HEK-293 and L929 cell lines, showed no significant toxicity compared to cancer cells, suggesting that the nanoformulations are safe for normal cells (Supplementary Materials, Table S1).

### 3.7. Cell Morphology Analysis

**Figure 7:**
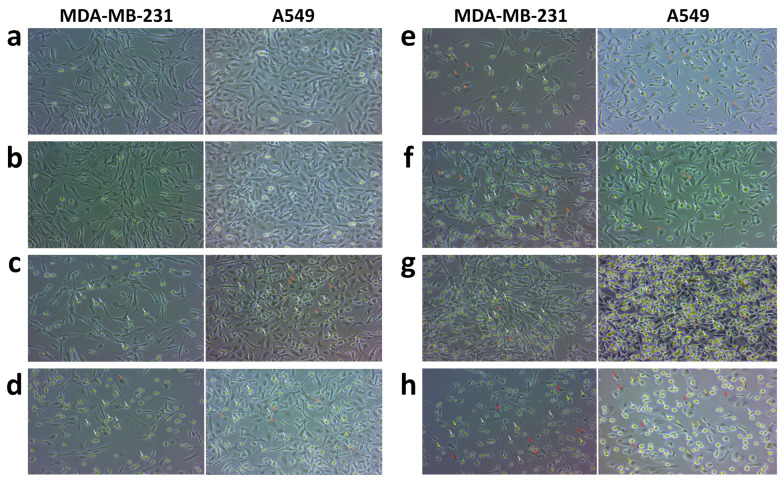
Morphological analysis by control, free drugs, and drug(s)-loaded nanoliposomes on MDA-MB-231 and A549 cell lines. (**a**) Control, (**b**) free liposomes, (**c**) free CDDP, (**d**) free DADS, (**e**) free CDDP/DADS, (**f**) Lipo-CDDP, (**g**) Lipo-DADS, and (**h**) Lipo-CDDP/DADS. White arrows indicate apoptotic cells and orange arrows indicate the bubbling of cells. The yellow arrow indicates chromatin condensation/blebs, and the red arrow indicates spikes in apoptotic undergoing cells.

Morphological analysis showed the morphological changes in MDA-MB-231 and A549 cells by Lipo-CDDP, Lipo-DADS, and Lipo-CDDP/DADS. The control cells retained the morphology and showed regular adherent properties compared to the treated cells. Very few morphological changes among free CDDP, free DADS, and free CDDP/DADS were observed (Figure 7c–e). In contrast, exposure of MDA-MB-231 and A549 cancer cells treated with Lipo-CDDP, Lipo-DADS, and Lipo-CDDP/DADS (Figure 7f–h) showed apoptotic features. The Lipo-CDDP/DADS show damaged cell membranes more than the single drug-loaded nanoliposomes. Figure 7 reveals the distorted membrane structure of the cancer cells. Drastic morphological changes were assessed in drug-treated cells in contrast to control cells.

### 3.8. DAPI Staining Analysis

**Figure 8:**
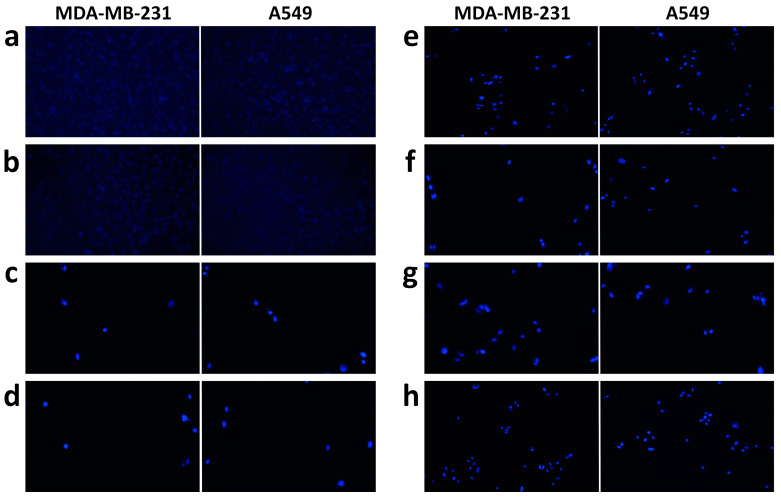
Nucleus changes due to cytotoxic effect by control, free drugs, and drug(s)-loaded nanoliposomes on MDA-MB-231 and A549 cell lines. (**a**) Control, (**b**) free liposomes, (**c**) free CDDP, (**d**) free DADS, (**e**) free CDDP/DADS, (**f**) Lipo-CDDP, (**g**) Lipo-DADS, and (**h**) Lipo-CDDP/DADS.

DAPI staining results showed nuclear changes in both cell lines. The untreated cells in both the cell lines were stained barely with blue fluorescence, whereas the treated cells emitted bright blue fluorescence. Figure 8 indicates the shape of the nucleus for untreated and treated cells. Significant and distinct nuclear changes were observed in Lipo-CDDP/DADS than their counterparts which are single drug-loaded nanoliposomes. The changes in nuclear morphology prove that Lipo-CDDP/DADS induce drug-mediated apoptosis, which in turn validates the results from the cytotoxicity analysis.

## 4. Discussion

Cancer is one of the deadliest diseases to date as it can affect almost all organs and metastasize nearby and distant organs, thereby increasing mortality. Classic therapies have proven ineffective due to various factors such as increased side effects, poor bioavailability, and low efficacy. In addition, the barrier formed by the endothelial cells in the vascular system prevents the transport of drugs in and out of the cells [15]. Moreover, most cytotoxic chemotherapeutic drugs are hydrophobic, which lessens their utilization in a cancer environment. To extinguish these shortcomings, nanoliposomal therapy has been used extensively. Co-encapsulation of synthetic and plant-derived compounds into the liposomes with different polarities can overcome the shortcomings mentioned above, where they were able to reduce the toxicity induced by synthetic drugs and target cancer in a precisely controlled manner [2,16,17].

Liposomes are biodegradable and biocompatible and can reduce drug toxicity and side effects. During the development of liposomes, numerous factors such as lipid, cholesterol composition, pH, temperature, etc., influence the particle size, which in turn affects the half-life of the liposomes, where they get eliminated from circulation by reticuloendothelial system and phagocytosis. As the lipid composition determines the liposome fluidity and surface characteristics, it also affects the ability to interact with cell membranes [18,19,20]. Liposome population properties include size, distribution, charge and permeability. Preparation methods, like sonication and extrusion, play a crucial role in regulating the size of the nanoformulation. Liposomal sizes determine whether liposomes can pass through cells’ vasculature. Our study achieved a nanoliposome size greater than 200 nm when sonication was done. Extrusion is needed as giant vesicles will not fit through leaky cell vasculature, so they must be sized accordingly. After extrusion, a nanoliposome size of less than 200 nm was achieved, which improved the half-life of the liposomes. In addition, it increases the enhanced permeability and retention effect and drug circulation time [21]. Here, in our liposomal formulation, we have used DLPC, a neutral lipid [22], to construct the liposomal formulation. A study reveals that neutral lipids positively impact the particle size, which might be due to the charge inclusion between the bilayer [23]. Usually, charged particles, i.e., anions and cations, have reduced solubility in the nonpolar phase, which decreases the rate of constant transfer, resulting in the charged liposomes being less permeable in the cell membrane [24].

Another factor that hinders the liposome formulation is cholesterol composition. Cholesterol has a great impact on the action of liposomes in a biological system as it plays a crucial role in liposome composition. This novel liposomal formulation includes cholesterol with the optimized lipid: cholesterol ratio of 10:2, which gives rigidity to the liposomal bilayer, thereby improving the physical stability of liposomes, which in turn facilitates increased encapsulation efficiency of both drugs into the liposomes. Through an encapsulation efficiency study, we infer that the formulation will be able to entrap a great percentage of drugs which would play an important role in drug delivery. As a result, we believe that the higher percentage of encapsulation was due to the hydrophilic and lipophilic nature of dual drugs, as the drugs did not need to compete against each other to occupy the bilayer space, where CDDP occupies the hydrophilic core and DADS occupies lipophilic bilayer [3,25].

The most challenging aspect of nanocarrier therapy is in vivo protein corona formation (protein binding) in the presence of charged particles [26]. The resident proteins in the biological fluid form an array called protein corona, come in contact with any foreign particle and eliminate it via antigen-presenting cells [27,28]. To overcome this issue, neutral liposomes were formulated in our study using neutral lipids [22]. DLPC, a neutral lipid, plays an important role in the surface charge of the formulated liposomes [29]. Further, the overall neutral charge of liposomes aids in the diffusion of drugs into the nonpolar interior of the lipid bilayer [30]. Our nanoliposomal formulation can reduce corona formation and increase the drug resident time in the tumor environment [27,31].

The zeta potential of our Lipo-CDDP/DADS was neutral, which was greatly influenced by choice of lipid. A study conducted by [32] concluded that neutral lipid exhibits a charge ranging between −10 to +10 mV conclusively, which was similar to our research. The PDI refers to the heterogeneity of particles based on the volume of a solution. PDI should be <0.7, which corresponds to the polydispersity of the samples [33]. Further, PDI < 0.1 is considered to be more monodispersed, which further reduces the aggregation or agglomeration of nanoformulation [34]. In accordance with the above statement, we have achieved PDI < 0.1 for Lipo-CDDP/DADS, which indicates its monodispersity of nanoliposomes in the solution.

The release profile of our study showed a rapid release of free DADS and free CDDP into the sink medium. However, Lipo-CDDP and Lipo-DADS showed a sustained release profile than the free drugs. There were no significant changes observed between the release pattern of nanoliposomal formulations. It may be because of the structure and composition of the liposomal membrane [35,36], which caused the drugs to release slowly into the sink medium [37]. A study by [38] showed a similar sustained release of arginylglycylaspartic acid (RGD)-modified curcumin-paclitaxel liposomes, further confirming our results.

Furthermore, in our previous study, we observed sustained release of gingerol-loaded DLPC liposomes, consistent with our study [39]. These findings show that the drug release pattern of liposomal formulations was due to diffusion, partitioning, and dissolution mechanisms [40]. This proves the sustained release and stable property of our nanoliposome formulation.

A study conducted by [41] discloses that an optimized concentration of stabilizing compounds, such as cholesterol, will lessen the bending elasticity of the liposomal membrane, which in turn maintains the stability of the nanoformulation. The colloidal stability assessment after storing nanoliposomal formulations at 4 °C reveals that the average size, zeta potential, PDI, and encapsulation efficiency have no drastic changes due to the lipid: cholesterol ratio and the attractive forces such as Vander Waals interaction and long-range electrostatic repulsion. The balance in these two forces and the formation of counter ions around the nanoliposomes, i.e., the electrical double layer, counterbalances the surface charge of our nanoliposomes resulting in strong stability of nanoformulation [19]. Further, the dissolution medium we used in our study was PBS (pH 7.4), which also enhances the stability of nanoliposomal formulation through steric stabilization interaction.

Our study evaluated the cytotoxic effect of free drugs and liposomal formulations on MDA-MB-231 and A549 cell lines. MDA-MB-231 cell line being aggressive cancer, exhibits metastatic pattern extending secondary breast cancer in lung, bone, liver, brain, etc., which results in poor survival of patients. The global cancer rate estimated by GLOBOCAN (2020) from Global Cancer Observatory (http://gco.iarc.fr, accessed on 15 December 2021) states that the most diagnosed cancer was lung cancer (11.4%), next to breast cancer (11.7%) [42,43]. 85% of lung cancer deaths are caused by non-small cell lung cancer, and the A549 cell line is the most commonly used cell line for non-small cell lung cancer studies. The proliferation of breast cancer stem cells leads to secondary primary lung cancer, which is highly dependent on common signaling pathways. Common signaling pathways such as the cell cycle, Toll-like receptor signaling pathway, Hippo signaling pathway, MAPK signaling pathway, PPAR signaling pathway, ERBB signaling pathway, AMPK signaling pathway, ERK signaling pathway, and microenvironmental factors such as extracellular matrix proteins, transforming growth factor-β, cancer-associated fibroblasts, etc., are associated with both breast cancer and lung cancer [44,45]. The CDDP and DADS being synthetic and plant-derived compounds, respectively plays a role in the up-regulation or down-regulation of the pathways mentioned above [46,47]. Also, the CDDP and DADS supposedly exert anti-cancer mechanisms involving some common genes and pathways such as p53, caspases family, AKT pathway, mTOR pathway, PI3K pathway, etc. [48]. Therefore, combining these two compounds may exert maximum inhibition of cancer metastasis and inhibits cancer cell growth [49].

The cytotoxicity assessment revealed that free drugs exert a cytotoxic effect with higher drug dose concentrations than their nanoliposomal counterparts. Compared to Lipo-CDDP and Lipo-DADS exhibited a good cytotoxic effect which explains the synthetic nature of the drug CDDP. In contrast, DADS, a plant-derived compound, requires concentration higher than the conventional drug [7,50,51]. The decrease in cell proliferation/growth was highest in dual drug-loaded nanoliposomes (Lipo-CDDP/DADS), which denotes that the drugs can internalize, interact, and accumulate cancer cells via transference or endocytosis [52,53,54]. This reveals the combinatorial effect of dual drug-loaded liposomes compared to the other treatment groups [55].

On the contrary, the free drugs and free CDDP/DADS exhibit cytotoxicity at higher concentrations on HEK-293 and L929 normal cells compared to the concentrations required to inhibit half the population of MDA-MB-231 and A549 cells. This may be due to enzyme inhibitors in normal cells that inhibit pharmacokinetics and shorten the duration of drug-cell interactions [56]. Similarly, the IC_50_ of HEK-293 and L929 cells treated with nanoliposome formulations are higher in comparison with MDA-MB-231 and A549 cells. The presence of tight junctions in the normal cells and the lack of ligand-receptor interactions in the cytomembrane infer the passing of drugs inside the cells. This could also be one of the hypothetical reasons for the low cytotoxic activity of free drugs and nanoformulations at lower drug concentrations against normal cells [57].

The obtained IC_50_ value through cytotoxicity assays was used for morphological study. The morphological assessment of both cell lines treated with free drugs and nanoliposomal formulations showed the structural changes exerted by them. The apoptotic characteristic of our nanoliposomal formulation showed significant structural changes compared to free drugs, with consequential changes in the shrinkage of cells, membrane distortion, and formation of pyknotic nuclei through chromatin condensation and echinoid spikes in the apoptosis-undergoing cells [58]. In addition, the cells undergoing apoptosis showed decreased cell adhesion properties to the well plate, which resulted in the floating of cells in the culture medium. And bubbling of some cells was also seen, which indicates cell death through necrosis [59].

DAPI staining revealed the morphological alterations of both cancer cell lines compared to the control. In our previous study, cells treated with nanoliposome formulation showed typical apoptotic characteristics of cell fragmentation and shrinkage, and also nucleus showed bright, uniform fluorescent, which indicates chromatin condensation [39]. The overall DAPI staining results show that apoptosis and cell membrane disintegration was mostly due to the action of dual drug-loaded nanoliposomes exerted on the cell lines.

Like most studies, the current study has limitations. Firstly, the current study examined the effects of nanoformulations on two cell lines, such as MDA-MB 231 and A549 cells. Additional studies using different breast and lung cancer cells further strengthen the translational value of these nanoformulations. Second, in this study, HEK-293 and L929 cells were used to assess offsite exposure and toxicity of free drugs and their nanoformulations. In contrast, normal breast epithelial and lung epithelial cell lines would be better suited to assess the direct effect of nanoformulations in the target organ. In addition, the present study examined the efficacy of lipo-CDDP/DADS in vitro. Therefore, in vivo studies are needed to determine the effect of lipo-CDDP/DADS on breast and lung cancer. Finally, to get a more comprehensive overview of the biological activity of lipo-CDDP/DADS, gene expression studies should be examined to determine the mechanism by which the nanoformulation acts against cancer cells.

## 5. Conclusions

This study concludes that the combinatorial effect of Lipo-CDDP/DADS has proven to be an effective remedy for treating cancers with improved bioavailability and stability. We also believe that this formulation would increase the on-site exposure of drugs, decreasing the damage exerted by anti-cancer drugs on normal cells. Comprehensively, this study proposes that the delivery of Lipo-CDDP/DADS could be a favorable therapeutic choice for treating various types of cancer.

## Figures and Tables

**Table 1:** Physical characterization of free liposome and drug-loaded liposomes.

No.	Formulation	Size	Zeta Potential	PDI
1	Free Liposome	94.39 nm	−1.01 mV	0.088
2	Lipo-CDDP	101.60 nm	−1.13 mV	0.038
3	Lipo-DADS	103.40 nm	−2.36 mV	0.015
4	Lipo-CDDP/DADS	105.50 nm	−1.34 mV	0.006

CDDP, cisplatin; DADS, diallyl disulfide; PDI, polydispersity index.

**Table 2:** Encapsulation efficiency of formulated nanoliposomes.

No.	Drug	Lipo-CDDP	Lipo-DADS	Lipo-CDDP/DADS
1	CDDP	90.21 ± 4.21%	-	80.24 ± 2.32%
2	DADS	-	93.14 ± 2.50%	89.02 ± 3.50%

CDDP, cisplatin; DADS, diallyl disulfide; PDI, polydispersity index; mean ± standard deviation (*n* = 3).

**Table 3:** Colloidal stability of Lipo-CDDP/DADS.

No.	Storage Period	Size	Zeta Potential	PDI	Encapsulation Efficiency	
					CDDP	DADS
1	Day 1	105.50 nm	−1.34 mV	0.006	80.15 ± 2.32%	89.23 ± 3.50%
2	Day 90	108.80 nm	−0.98 mv	0.040	78.45 ± 1.65%	85.31 ± 1.98%

CDDP, cisplatin; DADS, diallyl disulfide; PDI, polydispersity index; mean ± standard deviation (*n* = 3).

**Table 4:** IC_50_ values for free and nanoliposomal drugs on cancer cells by MTT assay.

No.	Drugs	MDA-MB-231	A549
1	Free CDDP	11.71 ± 1.50 μM	13.24 ± 1.21 μM
2	Free DADS	24.12 ± 1.20 μM	29.51 ± 0.98 μM
3	Free CDDP/DADS	15.78 ± 2.02 μM	11.25 ± 1.85 μM
4	Lipo-CDDP	5.74 ± 0.96 μM	6.25 ± 1.45 μM
5	Lipo-DADS	9.51 ± 1.30 μM	10.21 ± 2.21 μM
6	Lipo-CDDP/DADS	12.15 ± 0.45 μM	14.53 ± 1.67 μM

CDDP, cisplatin, DADS, diallyl disulfide, mean ± standard deviation (*n* = 3).

## Data Availability

The authors confirm that the data supporting this study can be found in the article and its Supplementary Materials.

## Supplementary Materials

The following supporting information can be downloaded at: https://www.mdpi.com/article/10.3390/biomedicines11041021/s1; Table S1: IC50 values for free drugs and nanoliposomal drugs on normal cell lines by MTT assay.

## References

[1] Hassan Y.A., Alfaifi M.Y., Shati A.A., Elbehairi S.E.I., Elshaarawy R.F.M., Kamal I. (2022). Co-delivery of anticancer drugs via poly (ionic crosslinked chitosan-palladium) nano capsules: Targeting more effective and sustainable cancer therapy. J. Drug Deliv. Sci. Technol..

[2] Hu C.M.J., Zhang L. (2012). Nanoparticle-based combination therapy toward overcoming drug resistance in cancer. Biochem. Pharmacol..

[3] Liu J., Wang Z., Li F., Gao J., Wang L., Huang G. (2015). Liposomes for systematic delivery of vancomycin hydrochloride to decrease nephrotoxicity: Characterization and evaluation. Asian J. Pharm. Sci. JPS.

[4] Franco M.S., Silva C.A., Leite E.A., Silveira J.N., Teixeira C.S., Cardoso V.N., Ferreira E., Cassali G.D., de Barros A.L.B., Oliveira M.C. (2021). Investigation of the antitumor activity and toxicity of cisplatin loaded pH-sensitive-pegylated liposomes in a triple negative breast cancer animal model. J. Drug Deliv. Sci. Technol..

[5] Callejo A., Sedó-Cabezón L., Juan I.D., Llorens J. (2015). Cisplatin-Induced Ototoxicity: Effects, Mechanisms and Protection Strategies. Toxics.

[6] Rediti M., Messina C. (2018). Towards treatment personalization in triple negative breast cancer: Role of platinum-based neoadjuvant chemotherapy. Breast Cancer Res. Treat..

[7] Abdel-Hamid N.M., Abass S.A., Eldomany R.A., Abdel-Kareem M.A., Zakaria S. (2022). Dual regulating of mitochondrial fusion and Timp-3 by leflunomide and diallyl disulfide combination suppresses diethylnitrosamine-induced hepatocellular tumorigenesis in rats. Life Sci..

[8] Abdel-Daim M.M., Abdel-Rahman H.G., Dessouki A.A., El-Far A.H., Khodeer D.M., Bin-Jumah M., Alhader M.S., Alkahtani S., Aleya L. (2020). Impact of garlic (Allium sativum) oil on cisplatin-induced hepatorenal biochemical and histopathological alterations in rats. Sci. Total Environ..

[9] Bulbake U., Doppalapudi S., Kommineni N., Khan W. (2017). Liposomal Formulations in Clinical Use: An Updated Review. Pharmaceutics.

[10] De Vita A., Liverani C., Molinaro R., Martinez J.O., Hartman K.A., Spadazzi C., Miserocchi G., Taraballi F., Evangelopoulos M., Pieri F. (2021). Lysyl oxidase engineered lipid nanovesicles for the treatment of triple negative breast cancer. Sci. Rep..

[11] Merino M.T.G., Lozano T., Casares N., Lana H., Trocóniz I.F., Hagen T.L.T., Kochan G., Berraondo P., Zalba S., Garrido M.C.D. (2021). Dual activity of PD-L1 targeted Doxorubicin immunoliposomes promoted an enhanced efficacy of the antitumor immune response in melanoma murine model. J. Nanobiotechnol..

[12] Su C.W., Chiang C.S., Li W.M., Hu S.H., Chen S.Y. (2014). Multifunctional nanocarriers for simultaneous encapsulation of hydrophobic and hydrophilic drugs in cancer treatment. Nanomedicine.

[13] Naderinezhad S., Amoabediny G., Haghiralsadat F. (2017). Co-delivery of hydrophilic and hydrophobic anticancer drugs using biocompatible pH-sensitive lipid-based nano-carriers for multidrug-resistant cancers. RSC Adv..

[14] Zhang H., D’Souza G.G.M. (2017). Thin-Film Hydration Followed by Extrusion Method for Liposome Preparation. Liposomes.

[15] Albanese A., Tang P.S., Chan W.C. (2012). The Effect of Nanoparticle Size, Shape, and Surface Chemistry on Biological Systems. Annu. Rev. Biomed. Eng..

[16] Lin S.R., Chang C.H., Hsu C.F., Tsai M.J., Cheng H., Leong M.K., Sung P.J., Chen J.C., Weng C.F. (2019). Natural compounds as potential adjuvants to cancer therapy: Preclinical evidence. Br. J. Pharmacol..

[17] Bama E.S., Grace V.M.B., Sundaram V., Jesubatham P.D. (2019). Synergistic effect of co-treatment with all-trans retinoic acid and 9-cis retinoic acid on human lung cancer cell line at molecular level. 3 Biotech.

[18] Lasic D.D. (1993). Liposomes: From Physics to Applications.

[19] Israelachvili J.N. (1992). Intermolecular and Surface Forces.

[20] Israelachvili J.N. (2011). Intermolecular and Surface Forces.

[21] Hwang K.J., Padki M.M., Chow D.D., Essien H.E., Lai J.Y., Beaumier P.L. (1987). Uptake of small liposomes by non-reticuloendothelial tissues. Biochim. Biophys. Acta.

[22] Liu J. (2016). Interfacing Zwitterionic Liposomes with Inorganic Nanomaterials: Surface Forces, Membrane Integrity, and Applications. Langmuir.

[23] Alhariri M., Majrashi M.A., Bahkali A.H., Almajed F.S., Azghani A.O., Khiyami M.A., Alyamani E.J., Aljohani S.M., Halwani M.A. (2017). Efficacy of neutral and negatively charged liposome-loaded gentamicin on planktonic bacteria and biofilm communities. Int. J. Nanomed..

[24] Nel A.E., Mädler L., Velegol D., Xia T., Hoek E.M.V., Somasundaran P., Klaessig F., Castranova V., Thompson M. (2009). Understanding bio physicochemical interactions at the nano–bio interface. Nat. Mater..

[25] Sciolla F., Truzzolillo D., Chauveau E., Trabalzini S., Di Marzio L., Carafa M., Marianecci C., Sarra A., Bordi F., Sennato S. (2021). Influence of drug/lipid interaction on the entrapment efficiency of isoniazid in liposomes for antitubercular therapy: A multi-faced investigation. Colloids Surf. B Biointerfaces.

[26] Monopoli M.P., Åberg C., Salvati A., Dawson K.A. (2012). Biomolecular coronas provide the biological identity of nanosized materials. Nat. Nanotechnol..

[27] Safavi-Sohi R., Maghari S., Raoufi M., Jalali S.A., Hajipour M.J., Ghassempour A., Mahmoudi M. (2016). Bypassing Protein Corona Issue on Active Targeting: Zwitterionic Coatings Dictate Specific Interactions of Targeting Moieties and Cell Receptors. ACS Appl. Mater. Interfaces.

[28] Rampado R., Crotti S., Caliceti P., Pucciarelli S., Agostini M. (2020). Recent Advances in Understanding the Protein Corona of Nanoparticles and in the Formulation of “Stealthy” Nanomaterials. Front. Bioeng. Biotechnol..

[29] Chotphruethipong L., Battino M., Benjakul S. (2020). Effect of stabilizing agents on characteristics, antioxidant activities and stability of liposome loaded with hydrolyzed collagen from defatted Asian sea bass skin. Food Chem..

[30] Singh V., Khullar P., Dave P.N., Kaur N. (2013). Micelles, mixed micelles, and applications of polyoxypropylene (PPO)-polyoxyethylene (PEO)-polyoxypropylene (PPO) triblock polymers. Int. J. Ind. Chem..

[31] García K.P., Zarschler K., Barbaro L., Barreto J.A., O’Malley W., Spiccia L., Stephan H., Graham B. (2014). Zwitterionic-Coated “Stealth” Nanoparticles for Biomedical Applications: Recent Advances in Countering Biomolecular Corona Formation and Uptake by the Mononuclear Phagocyte System. Small.

[32] Smith M.C., Crist R.M., Clogston J.D., McNeil S.E. (2017). Zeta potential: A case study of cationic, anionic, and neutral liposomes. Anal. Bioanal. Chem..

[33] Danaei M., Dehghankhold M., Ataei S., Hasanzadeh Davarani F., Javanmard R., Dokhani A., Khorasani S., Mozafari M.R. (2018). Impact of Particle Size and Polydispersity Index on the Clinical Applications of Lipidic Nanocarrier Systems. Pharmaceutics.

[34] Truzzi E., Capocefalo A., Meneghetti F., Maretti E., Mori M., Iannuccelli V., Domenici F., Castellano C., Leo E. (2022). Design and physicochemical characterization of novel hybrid SLN-liposome nanocarriers for the smart co-delivery of two antitubercular drugs. J. Drug Deliv. Sci. Technol..

[35] Huang J., Wang Q., Chu L., Xia Q. (2020). Liposome-chitosan hydrogel bead delivery system for the encapsulation of linseed oil and quercetin: Preparation and in vitro characterization studies. LWT.

[36] Wong P.T., Choi S.K. (2015). Mechanisms of Drug Release in Nanotherapeutic Delivery Systems. Chem. Rev..

[37] Xie Q., Deng W., Yuan X., Wang H., Ma Z., Wu B., Zhang X. (2018). Selenium-functionalized liposomes for systemic delivery of doxorubicin with enhanced pharmacokinetics and anticancer effect. Eur. J. Pharm. Biopharm..

[38] Jiang K., Shen M., Xu W. (2018). Arginine, glycine, aspartic acid peptide-modified paclitaxel and curcumin co-loaded liposome for the treatment of lung cancer: In vitro/vivo evaluation. Int. J. Nanomed..

[39] Thangavelu P., Sundaram V., Gunasekaran K., Mujyambere B., Raju S., Kannan A., Arasu A., Krishna K., Ramamoorthi J., Ramasamy S. (2022). Development of optimized novel liposome loaded with 6-gingerol and assessment of its therapeutic activity against NSCLC In vitro and In vivo experimental models. Chem. Phys. Lipids.

[40] Español L., Larrea A., Andreu V., Mendoza G., Arruebo M., Sebastian V., Aurora-Prado M.S., Kedor-Hackmann E.R.M., Santoro M.I.R.M., Santamaria J. (2016). Dual encapsulation of hydrophobic and hydrophilic drugs in PLGA nanoparticles by a single-step method: Drug delivery and cytotoxicity assays. RSC Adv..

[41] Ullmann K., Leneweit G., Nirschl H. (2021). How to Achieve High Encapsulation Efficiencies for Macromolecular and Sensitive APIs in Liposomes. Pharmaceutics.

[42] Sung H., Ferlay J., Siegel R.L., Laversanne M., Soerjomataram I., Jemal A., Bray F. (2021). Global Cancer Statistics 2020: GLOBOCAN Estimates of Incidence and Mortality Worldwide for 36 Cancers in 185 Countries. CA Cancer J. Clin..

[43] Wennstig A.K., Wadsten C., Garmo H., Johansson M., Fredriksson I., Blomqvist C., Holmberg L., Nilsson G., Sund M. (2021). Risk of primary lung cancer after adjuvant radiotherapy in breast cancer-a large population-based study. NPJ Breast Cancer.

[44] Jin L., Han B., Siegel E., Cui Y., Giuliano A., Cui X. (2018). Breast cancer lung metastasis: Molecular biology and therapeutic implications. Cancer Biol. Ther..

[45] Wang H., Zhang G., Zhang H., Zhang F., Zhou B., Ning F., Wang H.S., Cai S.H., Du J. (2014). Acquisition of epithelial–mesenchymal transition phenotype and cancer stem cell-like properties in cisplatin-resistant lung cancer cells through AKT/β-catenin/Snail signaling pathway. Eur. J. Pharmacol..

[46] Romera-Giner S., Andreu Martínez Z., García-García F., Hidalgo M.R. (2021). Common pathways and functional profiles reveal underlying patterns in Breast, Kidney and Lung cancers. Biol. Direct.

[47] Song X., Yue Z., Nie L., Zhao P., Zhu K., Wang Q. (2021). Biological Functions of Diallyl Disulfide, a Garlic-Derived Natural Organic Sulfur Compound. Evid. Based Complement. Alternat. Med..

[48] Wu Y.R., Li L., Sun X.C., Wang J., Ma C.Y., Zhang Y., Qu H.L., Xu R.X., Li J.J. (2021). Diallyl disulfide improves lipid metabolism by inhibiting PCSK9 expression and increasing LDL uptake via PI3K/Akt-SREBP2 pathway in HepG2 cells. Nutr. Metab. Cardiovasc. Dis..

[49] Cui Z., Li D., Zhao J., Chen K. (2022). Falnidamol and cisplatin combinational treatment inhibits non-small cell lung cancer (NSCLC) by targeting DUSP26-mediated signal pathways. Free Radic. Biol. Med..

[50] Roy A. (2021). Plumbagin: A Potential Anti-Cancer Compound. Mini Rev. Med. Chem..

[51] Amini Z., Rudsary S.S., Shahraeini S.S., Dizaji B.F., Goleij P., Bakhtiari A., Irani M., Sharifianjazi F. (2021). Magnetic bioactive glasses/Cisplatin loaded-chitosan (CS)-grafted- poly (ε-caprolactone) nanofibers against bone cancer treatment. Carbohydr. Polym..

[52] Choi E.J., Kim G.H. (2009). Apigenin causes G2/M arrest associated with the modulation of p21Cip1 and Cdc2 and activates p53-dependent apoptosis pathway in human breast cancer SK-BR-3 cells. J. Nutr. Biochem..

[53] Pagano R.E., Weinstein J.N. (1978). Interactions of liposomes with mammalian cells. Annu. Rev. Biophys. Bioeng..

[54] Anuje M., Sivan A., Khot V.M., Pawaskar P.N., Thorat N.D., Bauer J. (2020). Cellular interaction and toxicity of nanostructures. Nanomedicines for Breast Cancer Theranostics.

[55] Lu X., Lu X., Yang P., Zhang Z., Lv H. (2022). Honokiol nanosuspensions loaded thermosensitive hydrogels as the local delivery system in combination with systemic paclitaxel for synergistic therapy of breast cancer. Eur. J. Pharm. Sci..

[56] Vigneron N. (2015). Human Tumor Antigens and Cancer Immunotherapy. BioMed. Res. Int..

[57] Hernandez-Delgadillo R., García-Cuellar C.M., Sánchez-Pérez Y., Pineda-Aguilar N., Martínez-Martínez M.A., Rangel-Padilla E.E., Nakagoshi-Cepeda S.E., Solís-Soto J.M., Sánchez-Nájera R.I., Nakagoshi-Cepeda M.A.A. (2018). In vitro evaluation of the antitumor effect of bismuth lipophilic nanoparticles (BisBAL NPs) on breast cancer cells. Int. J. Nanomed..

[58] Abdel Wahab S.I., Abdul A.B., Alzubairi A.S., Mohamed Elhassan M., Mohan S. (2009). In Vitro Ultramorphological Assessment of Apoptosis Induced by Zerumbone on (HeLa). J. Biomed. Biotechnol..

[59] Syed Abdul Rahman S.N., Abdul Wahab N., Abd Malek S.N. (2013). In Vitro Morphological Assessment of Apoptosis Induced by Antiproliferative Constituents from the Rhizomes of *Curcuma zedoaria*. Evid. Based Complement. Alternat. Med..

