# Supplementary material for: Cytotoxic Effects of Nanoliposomal Cisplatin and Diallyl Disulfide on Breast Cancer and Lung Cancer Cell Lines
Source: Biomedicines. 2023 Mar 27;11(4):1021. doi: 10.3390/biomedicines11041021 (PMC10136257; doi:10.3390/biomedicines11041021)
Supplement: Supplementary file 1 [file biomedicines-11-01021-s001.zip › biomedicines-2266209-supplementary.pdf]

Table S1. IC_{50} values for free drugs and nanoliposomal drugs on normal cell lines by MTT assay.

No.	Drugs	HEK-293	L929
1	Free CDDP	22.21 ± 2.60 μM	25.82 ± 1.21 μM
2	Free DADS	35.34 ± 1.34 μM	38.95 ± 2.40 μM
3	Free CDDP/DADS	18.19 ± 3.02 μM	21.13 ± 0.92 μM
4	Lipo-CDDP	10.41 ± 1.54 μM	12.62 ± 1.29 μM
5	Lipo-DADS	17.65 ± 0.94 μM	19.48 ± 1.84 μM
6	Lipo-CDDP/DADS	25.54 ± 1.72 μM	35.12 ± 2.01 μM

CDDP, cisplatin; DADS, diallyl disulfide; mean ± standard deviation ($n = 3$)
